# Water-induced bridgmanite coarsening may produce highly viscous lower mantle seismic anomalies

**DOI:** 10.1093/nsr/nwag464

**Published:** 2026-07-31

**Authors:** Yifu Lyu, Hongzhan Fei, Amrita Chakraborti, Maxim Ballmer, Ulrich Faul, Shan Zhou, Yan Yang, Jia Liu, Fang Xu, Suyu Fu, Baohua Zhang, Qunke Xia, Tomoo Katsura

**Affiliations:** Research Center for Earth and Planetary Material Sciences, School of Earth Sciences, Zhejiang University, Hangzhou 310058, China; Research Center for Earth and Planetary Material Sciences, School of Earth Sciences, Zhejiang University, Hangzhou 310058, China; Bayerisches Geoinstitut, University of Bayreuth, Bayreuth 95440, Germany; Unité Matériaux et Transformations, Université de Lille, Lille 59000, France; Department of Earth Sciences, University College London, London WC1E 6BT, UK; Earth Atmospheric and Planetary Sciences, Massachusetts Institute of Technology, Cambridge, MA 02139, USA; Research Center for Earth and Planetary Material Sciences, School of Earth Sciences, Zhejiang University, Hangzhou 310058, China; Research Center for Earth and Planetary Material Sciences, School of Earth Sciences, Zhejiang University, Hangzhou 310058, China; Research Center for Earth and Planetary Material Sciences, School of Earth Sciences, Zhejiang University, Hangzhou 310058, China; Research Center for Earth and Planetary Material Sciences, School of Earth Sciences, Zhejiang University, Hangzhou 310058, China; Research Center for Earth and Planetary Material Sciences, School of Earth Sciences, Zhejiang University, Hangzhou 310058, China; Research Center for Earth and Planetary Material Sciences, School of Earth Sciences, Zhejiang University, Hangzhou 310058, China; Research Center for Earth and Planetary Material Sciences, School of Earth Sciences, Zhejiang University, Hangzhou 310058, China; Bayerisches Geoinstitut, University of Bayreuth, Bayreuth 95440, Germany

**Keywords:** bridgmanite, grain growth, water effect, viscosity, lower mantle, LLVPs

## Abstract

Two seismic anomalies, known as large low velocity provinces (LLVPs), are detected seismically at the bottom of the Earth’s lower mantle. Acting as the source of mantle plumes and as barriers to mantle convection, LLVPs have stayed in the lower mantle over a long duration and significantly influence the dynamics of the Earth. However, it is unclear how the LLVPs could be preserved in the lower mantle because the elevated temperature and water content within LLVPs should reduce their rheological strength, leading to a rapid mixing by mantle convection. Here, through high-pressure experiments, we demonstrate that the grain growth of bridgmanite, the dominant mineral in the lower mantle, is significantly enhanced by water incorporation. The relatively water-rich conditions of LLVPs and consequent larger grain size may cause their viscosity 1.4 orders of magnitude higher than the surrounding mantle despite the negatively thermo-hydrous effect on mineral strength, providing a reliable mechanism for their longevity.

## Teaser

Water promotes grain growth in bridgmanite, potentially increasing the viscosity of giant seismic anomalies in the Earth’s lower mantle and helping maintain their long-term stability.

## INTRODUCTION

Two large structures with thousands of kilometers in width and up to 1800 km in height, known as large low velocity provinces (LLVPs), have been identified just above the core-mantle boundary beneath the Pacific Ocean and Africa by global seismic tomography [1]. They are mainly considered as thermochemical anomalies characterized by low seismic velocity, low attenuation [2] and high density compared to the surrounding mantle [3–5]. The LLVPs not only act as sources of upwelling plumes, resulting in intraplate volcanism such as large igneous provinces (LIPs) and kimberlite eruptions, but also as barriers to mantle convection, leading to the heterogeneity of the lower mantle [3,6,7]. Investigating the dynamics of LLVPs is therefore crucial for understanding the convection and chemical evolution of the mantle.

Although the exact origin of LLVPs is under debate, they are widely hypothesized to be formed either by the preservation of primordial materials during magma ocean solidification or accumulation of oceanic crusts from slab subduction because both can match the low seismic wave velocity of LLVPs [8–11]. However, both models require the assumption that the LLVPs should be relatively immobile compared to the convective mantle [12,13] and preserved over long duration without mixing by mantle convection, which is also supported by the distributions of LIPs and kimberlite eruptions [7]. Thus, the viscosity of materials in LLVPs should be higher than the surrounding mantle for the preservation against mantle convection.

On the other hand, the low seismic velocity and the origination of mantle plumes suggest a temperature condition of LLVPs 200–400 K higher (varying with depth) than that of the surrounding mantle [14,15], while the relatively high water contents observed in LIPs suggest the water-enriched sources linked to LLVPs [16] despite of the remaining uncertainty about whether LLVPs are systematically water-rich or not. Sequentially, both thermal and water weakening of rocks may reduce the rheological strength of LLVPs, causing them to be mixed rapidly during mantle convection rather than stabilized near the core-mantle boundary over a long history since the density anomaly alone is insufficient to sustain the longevity of the LLVPs [5,17,18].

Clearly, to coordinate the features of thermochemical anomaly and long-term preservation, materials in the LLVPs should be rheologically strong (high viscosity) even under high temperature and water-rich conditions. Upon the origination, the LLVPs could be enriched with davemaoite (subducted oceanic crust scenario) or Fe^3+^-bridgmanite (ancient materials scenario). However, both Fe^3+^-rich bridgmanite and davemaoite may further cause the LLVPs rheologically weaker than the surrounding mantle [19,20]. Thus, the question of how LLVPs maintain their rheological strength remains unresolved.

Grain size (*d*) is an important parameter controlling lower mantle viscosity [21,22]. The general absence of seismic anisotropy in most regions of the lower mantle suggests the operation of diffusion creep [23,24], in which viscosity increases strongly with grain size [2,25,26]. Considering that the grain growth of shallow mantle minerals such as olivine and wadsleyite is enhanced significantly by temperature and water [27–29], the potential large grain sizes of bridgmanite in LLVPs due to the high temperature and water-rich conditions may play a key role in maintaining their stability.

Here, to constrain the stability of LLVPs, we investigated the grain growth rate of bridgmanite coexisting with ferropericlase (dominant and secondary minerals in the lower mantle, respectively) as a function of water content by high-pressure and high-temperature multi-anvil experiments under lower mantle conditions at 25 GPa, 2000 K, with water contents in the samples varied by the amounts of water sources (MgO-Mg(OH)_2_) and durations for 10–500 min (Fig. S1 and Table S1). Combined with the temperature dependence we determined previously [30], the viscosity contrast between LLVPs and surrounding mantle was estimated based on the grain-size-controlled rheology. Finally, our results provide a helpful mechanism for the relative immobility of LLVPs.

## RESULTS AND DISCUSSION

### The effect of water on the growth rate

**Figure 1.**
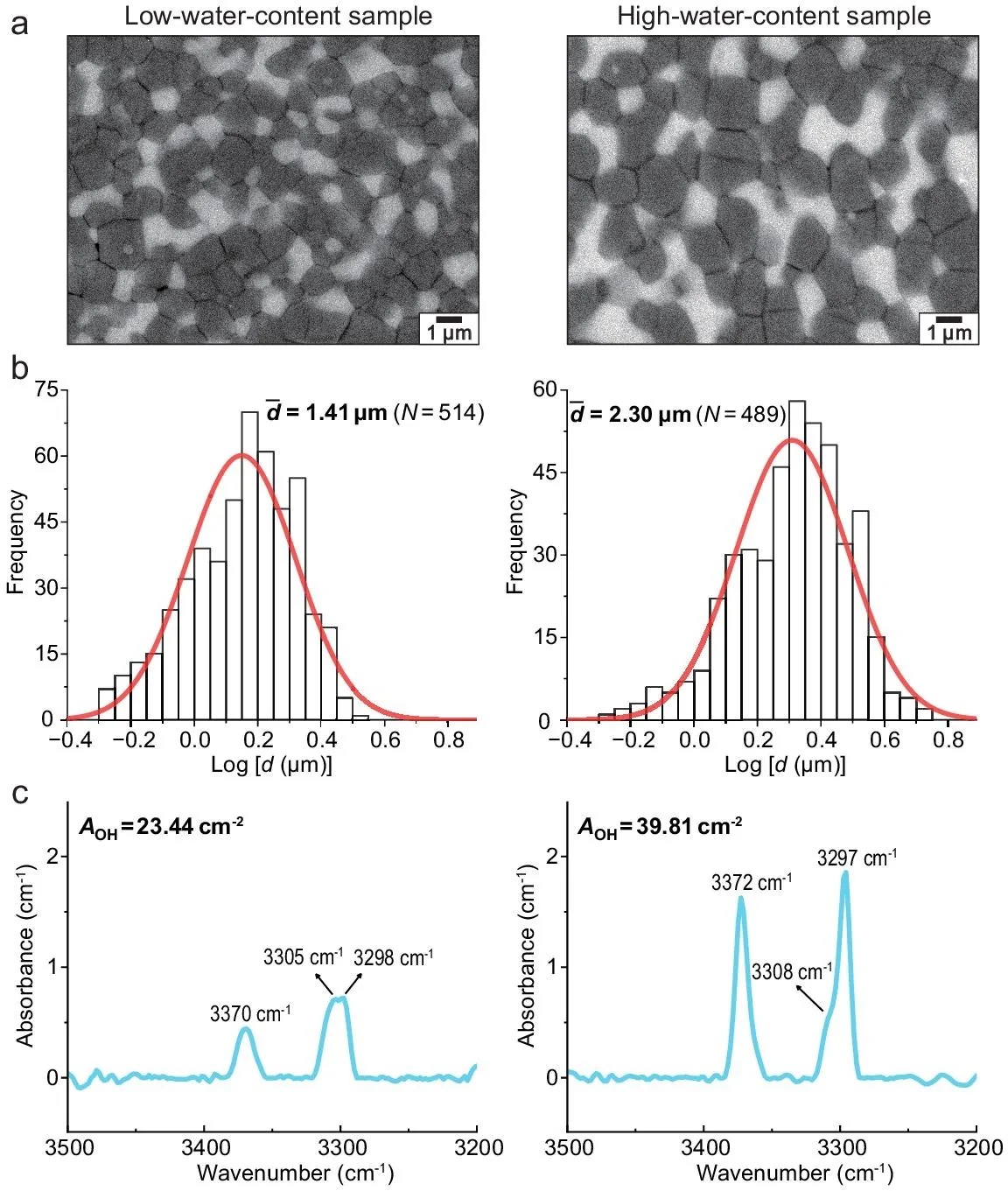
Grain sizes of bridgmanite with varying water contents after growth at 25 GPa and 2000 K for 30 min. (a) BSE images. (b) Grain-size distribution. $\bar{d}$: the average grain size of the sample. *N*: number of bridgmanite grains for statistical analysis. (c) Infrared absorptions of periclase (*A*_OH_), which reflect the relative changes of water contents in the samples (see Methods).

The experimental details are given in the Methods section. In the run products, bridgmanite and ferropericlase grains with regular shapes were clearly distinguished by brightness contrast, while sharp grain boundaries were identified. Similar with dry condition experiments [30], triple junctions with 120-degree angles can be clearly defined in the backscattered electron (BSE) images (Fig. 1). No compositional heterogeneity was found in the bridgmanite–ferropericlase aggregates. Both the MgO + Mg(OH)_2_ and MgO layers show a single phase of MgO with grain sizes of several to tens of microns. Melt phase was not detected in the run products, indicating the water-undersaturated conditions because the presence of melt will affect the grain growth mechanism and thus the bulk water contents in the experiments were controlled at extremely low levels (less than a few thousand wt. ppm) to avoid the formation of hydrous melt (see Methods).

The grain size of bridgmanite in logarithmic units (log *d*) generally follows a Gaussian distribution (Fig. 1 and Fig. S2), although a few samples show slight skewness, which is likely attributable to statistical uncertainty. Grain sizes across different regions of each sample show a uniform distribution (Fig. S3). As expected, samples containing more water (higher absorbance in infrared spectroscopy analysis) show systematically larger grain sizes of both bridgmanite and ferropericlase grains (Fig. 1 and Fig. S1), indicating a significant enhancement of grain growth rate by water incorporation.

For polycrystalline aggregates, grain growth follows a power-law relationship,


(1) \begin{eqnarray*}
{d}_t^n - d_0^n = kt,
\end{eqnarray*}

where *t* is the growth duration, *d*_0_ and *d*_t_ are the grain sizes of the samples initially and after a heating duration of *t*, respectively, *k* is the temperature- and water-content-dependent growth rate and *n* is the grain-size exponent.

Since *d*_t_ evolves with time ($\frac{{\partial ( {{\mathrm{log}}d} )}}{{\partial ( {{\mathrm{log}}t} )}} = \frac{1}{n}$), time series experiments are essential for investigating grain growth kinetics. This has been conducted in our recent studies, showing a grain-size exponent *n* of 5.2 ± 0.3 for bridgmanite in the bridgmanite–ferropericlase system under dry condition [21,30]. Although the effect of water on *n* is unknown, it is expected to be negligibly small because of these reasons: (i) *n* is primarily controlled by number of phases in the system (*n* ≈ 2∼3 for single-phase system and *n* ≈ 5 for multiple-phase system) [31]; (ii) *n* is insensitive to chemical composition (MgSiO_3_–bridgmanite with 0% and 40% of FeAlO_3_ component have identical *n* [32]) or mineral types (different minerals such as forsterite–enstatite, forsterite–nickel and bridgmanite–ferropericlase systems have similar *n* [27,30,33]) and (iii) water does not meaningfully affect *n* for olivine [28]. While the water content in bridgmanite is limited [34], a single value of *n* (5.2 ± 0.3) determined for dry bridgmanite growth in the bridgmanite–ferropericlase system is used in this study.

As a chemically controlled kinetic process, *k* can be expressed as


(2) \begin{eqnarray*}
k = {k}_0f_{{{\mathrm{O}}}_2}^pa_{{\mathrm{MO}}}^qC_{{\mathrm{OH}}}^r{\mathrm{exp}}\left( { - \frac{{Q + P{V}^{\mathrm{*}}}}{{RT}}} \right),
\end{eqnarray*}

where *k*_0_ is the pre-exponential factor, ${f}_{{{\mathrm{O}}}_2}$ is oxygen fugacity (correlated with Fe^3+^ content), ${a}_{{\mathrm{MO}}}$ is activity of a metal oxide, *C*_OH_ is water content, *Q* is activation energy, V* is activation volume, *R* is the gas constant, *T* is temperature, and *p, q* and *r* are the exponents for ${f}_{{{\mathrm{O}}}_2}$, ${a}_{{\mathrm{MO}}}$ and *C*_OH_, respectively.

To constrain the water effect on grain growth rate (water exponent *r*) independently, all other parameters (*P, T*, ${a}_{{\mathrm{MO}}}$ and *f*_O2_) should be fixed while *C*_OH_ should be varied in experiments. The *P* and *T* are controlled experimentally with uncertainty of ∼1 GPa and 50∼ 100 K, respectively, for typical multi-anvil technique, while the coexistence with ferropericlase suggests a fixed ${a}_{{\mathrm{MO}}}$. The *f*_O2_ was theoretically not fixed in this study because of the possible oxidization of Fe^2+^ to Fe^3+^ by water. Nevertheless, even if all Fe^2+^ is oxidized and forms FeFeO_3_ component, the FeFeO_3_ concentration in bridgmanite will be <2 mol% according to the Fe contents given by electron probe microanalyzer (EPMA; Table S2). This would lead to a maximum effect of 0.11 on log*d* [32], which is within experimental uncertainty. Therefore, the effect of possible variation in *f*_O2_ (Fe^3+^) is expected to be limited in this study.

Note that the grain growth kinetics of bridgmanite is controlled by migration of grain boundaries, therefore, not affected by water within the grain interiors, but by grain-boundary water, which is practically difficult for quantitative characterization. Moreover, determining water content in bridgmanite remains debated [34–36]. However, water should be distributed in bridgmanite, coexisting (ferro)periclase and grain boundaries following their partition coefficients, while the partition coefficients can be assumed to be constant under a given thermodynamic condition (pressure, temperature and phase assemblages). More specifically, water contents on grain boundaries and grain interiors follow the equation [37–39],


(3) \begin{eqnarray*}
\frac{{{X}_{{\mathrm{GB}}}}}{{{X}_{{\mathrm{GB}}0} - {X}_{{\mathrm{GB}}}}} = \frac{{{X}_{{\mathrm{GM}}}}}{{1 - {X}_{{\mathrm{GM}}}}}\exp \left( {\frac{\gamma }{{RT}}} \right),
\end{eqnarray*}

where *X*_GB_ and *X*_GM_ are the water contents on grain boundaries and grain interiors, respectively, and *X*_GB0_ is the saturation level of *X*_GB_.

Within a limited range of bulk water content in the system, we have *X*_GB_ << *X*_GB0_ and *X*_GM_ << 1. Thus, the ratio *X*_GB_/*X*_GM_ (partitioning coefficient) is constant under the given *P–T* conditions. Consequently, we can evaluate the water effect on the grain growth of bridgmanite based on the relative change of water content in the coexisting periclase (*C*_OH_),


(4) \begin{eqnarray*}
k \propto {\left( {{C}_{{\mathrm{OH}}}} \right)}^r \propto {\left( {{A}_{{\mathrm{OH}}}} \right)}^r.
\end{eqnarray*}

Additionally, the *C*_OH_ is linearly proportional to the total infrared absorptions (*A*_OH_) based on the Beer–Lambert law, which can be quantitatively determined by infrared spectroscopy. Therefore, the absolute value of *C*_OH_ is unnecessary for determining the water-content exponent *r.*

**Figure 2.**
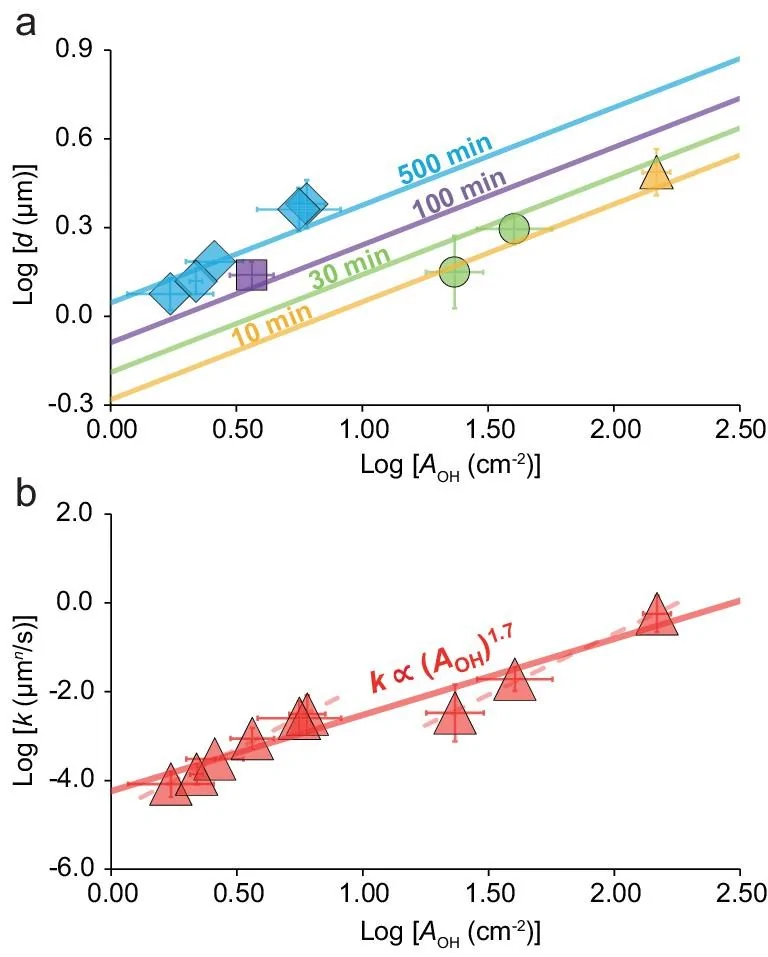
The effect of water on grain growth of bridgmanite. (a) Grain size of bridgmanite after growth at 25 GPa and 2000 K with different annealing durations. (b) Grain growth rate at 25 GPa and 2000 K as a function of water content. The *A*_OH_ refers to the infrared absorptions of MgO, which represent the relative change of water content of bridgmanite (detailed in the Methods). Horizontal error bars represent uncertainties of the measured *A*_OH_. Vertical error bars represent experimental uncertainties estimated from regression dispersion diagnostics, which mostly represent the experimental (pressure and temperature) uncertainty. Note that the data points in panel (b) could apparently fit separately for log(*A*_OH_) <1 and >1 (dashed lines), which give even steeper water dependence. However, the single-trend fit is retained for discussion in this study because the two-trend pattern is probably caused by experimental uncertainty.

Following the above strategy, the growth rate *k* is calculated from Equation (1) using *d*_t_ in the recovered samples by considering *d*_t_^n^ >> *d*_0_^n^ (see Methods). The water exponent *r* = 1.7 ± 0.2 is obtained by least-squares fitting of the *k* and *A*_OH_ data to Equation (4) (Fig. 2 and Table S1), constrained by the systematic trend across the full dataset. The large *r* value demonstrates a significant enhancement of bridgmanite grain growth by water.

From the perspective of defect chemistry, grain growth is typically limited by diffusion of the slowest species, which is silicon in bridgmanite [40,41]. It is therefore controlled by the concentration of point defects on Si sites ([V_Si_]). Although [V_Si_] on the grain boundaries of bridgmanite is unknown, it is expected to increase dramatically by adding water. In particular, if [V_Si_] is incorporated with three protons, its water-content exponent will be 3/2 or 5/3 (see ref. [42]), which aligns well with the exponent for grain growth.

### Water-induced grain-size coarsening in LLVPs

The LLVPs are formed primarily by accumulation of subducted materials or the preservation of ancient materials [3], both of which are expected to be water-rich because of the water-rich oceanic crusts [43] and the preferential partitioning of water in melt during magma ocean solidification [44,45], respectively. The geochemical data of LIPs originated from the mantle plumes suggest that their sources could have ∼2000 wt. ppm or even higher water [16,46]. In contrast, the surrounding lower mantle should contain much less water (50 wt. ppm or less) [47] if it is recycled by the subducted lithospheric mantle [48]. Namely, the water content in the LLVPs is expected to be >1.6 orders of magnitude larger than the surrounding mantle.

**Figure 3.**
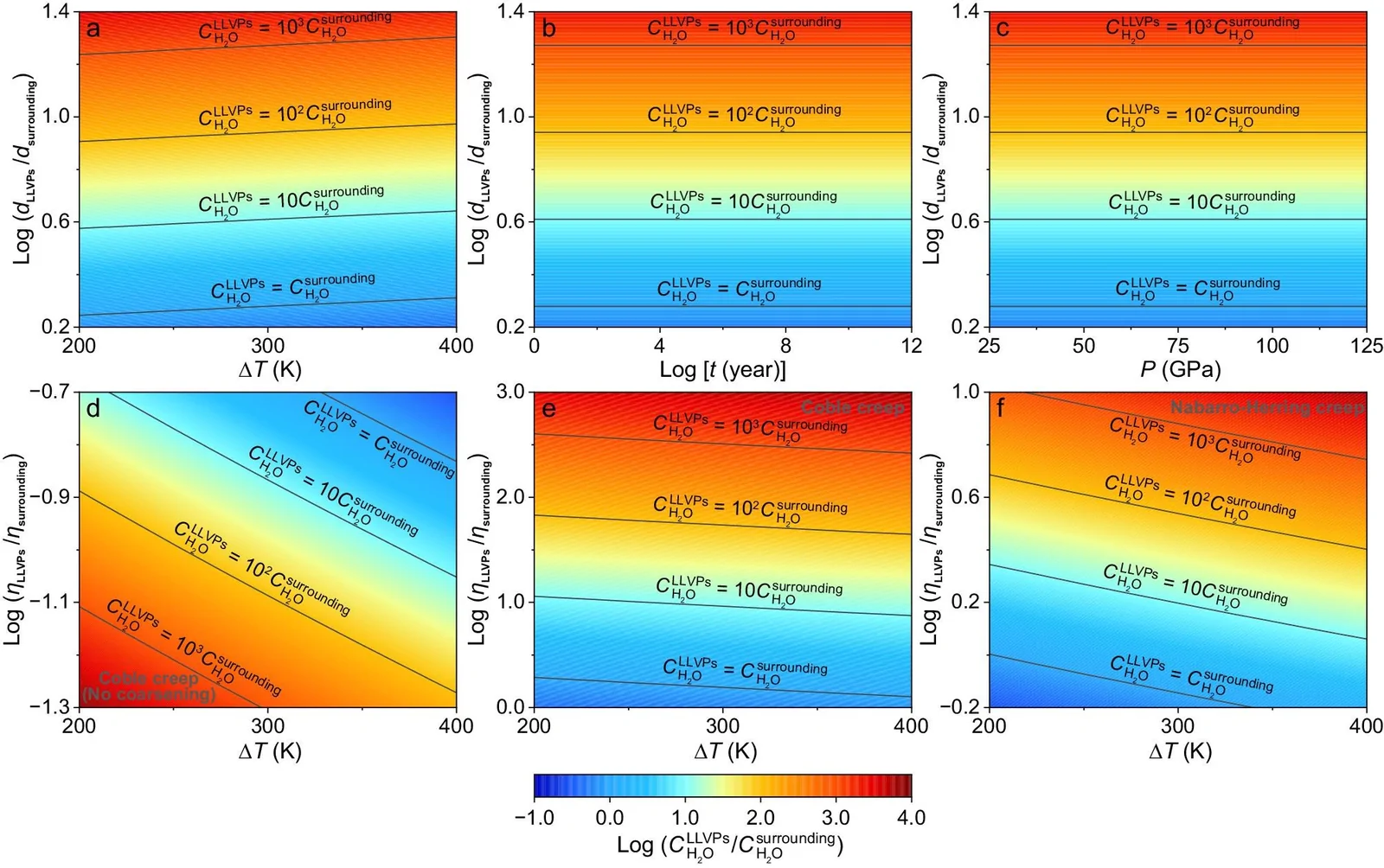
The grain-size contrast (*d*_LLVPs_/*d*_surrounding_) and viscosity contrast (*η*_LLVPs_/*η*_surrounding_) between LLVPs and surrounding mantle upon various parameters. (a) *d*_LLVPs_/*d*_surrounding_ as a function of temperature difference (Δ*T*) at various water-content (*C*_H2O_) conditions. (b) *d*_LLVPs_/*d*_surrounding_ as a function of geological time. (c) *d*_LLVPs_/*d*_surrounding_ as a function of pressure. (d) *η*_LLVPs_/*η*_surrounding_ as a function of Δ*T* at various *C*_H2O_ conditions in the Coble creep regime without considering the grain-size effects [53]. (e) *η*_LLVPs_/*η*_surrounding_ as a function of Δ*T* at various *C*_H2O_ conditions in the Coble creep regime [53]. (f) *η*_LLVPs_/*η*_surrounding_ as a function of Δ*T* at various *C*_H2O_ conditions in the Nabarro–Herring creep regime [54]. Note that the surrounding mantle is assumed to follow an adiabatic geotherm of 2500 K at a depth of 2400 km [49], and Δ*T* denotes the thermal excess of LLVPs relative to surrounding mantle [14,15]. In panels (b) and (c), Δ*T* is fixed at 300 K. A 10% enrichment of ferric iron in LLVPs is also included [10]. For the viscosity calculations, the weakening effects of both temperature [40] and water [63,64] are incorporated in addition to the grain-size effect.

Based on the significant water enhancement of bridgmanite grain growth demonstrated in this study and the large water-content contrast between LLVPs and the surrounding mantle as discussed above, bridgmanite in LLVPs should have much larger grain sizes than the surrounding mantle. Meanwhile, the ferric iron enrichment in LLVPs will also enlarge the grain-size contrast [10,32]. Using an adiabatic geotherm of 2500 K for the surrounding mantle [49] and 300 K higher in LLVPs [14,15] at 2400 km depth, and a 10% enrichment of ferric iron in LLVPs [10], the grain-size contrast between LLVPs and the surrounding mantle will reach 0.8 orders of magnitude over a short timescale (e.g. 100 Myr, Fig. 3a and b) [2].

The initial grain sizes of zero are assumed for the above calculation. This is reasonable for the oceanic crust accumulation scenario for LLVPs since grain-size reduction to zero occurs by phase transition across the 660-km discontinuity during subduction. For the ancient material scenario, the grain-size contrast between LLVPs and surrounding mantle will be even larger when considering the crystallization in magma ocean [24,25], the bridgmanite enrichment in LLVPs (lower ferropericlase fractions than the surrounding mantle) [21,24,26] and the grain-size reduction of the surrounding mantle during phase transitions driven by mantle convection [25,26,30] although in this case the contribution of water to the grain-size contrast will be smaller. Thus, the above calculation by assuming the initial grain sizes of zero represents a minimum grain-size contrast between LLVPs and surrounding mantle.

Because the bridgmanite grain growth rate and its water effect were determined in this study only at a pressure condition of 25 GPa, much lower than that of the LLVPs (∼1800–2900 km depths, equivalent to ∼80–130 GPa), while the pressure effect on the grain growth rate is unknown, it is difficult to estimate the absolute values of grain sizes in the LLVPs and surrounding mantle directly. However, pressure is expected to only affect grain-size variation with depth, while the relative difference between LLVPs and surrounding mantle at the same depth should not be affected (Fig. 3c). Additionally, the extrapolation from experimental grain growth duration to geological timescale may also cause uncertainty to the estimation of grain size in the lower mantle. But this should not affect the grain-size contrast between LLVPs and surrounding mantle either by considering that they have identical grain-size exponent *n* (Fig. 3b). Meanwhile, the uncertainty of the timescale extrapolation should be negligibly small because of the extremely large *n* (Fig. S6), i.e. small slope of the grain size versus duration curve ($\frac{{\partial ( {{\mathrm{log}}d} )}}{{\partial ( {{\mathrm{log}}t} )}} = \frac{1}{n}$).

### Implications for the LLVPs longevity and slab dynamics

The absence of pronounced seismic anisotropy in the lower mantle, at least in the regions far from subducting slabs where the differential stress is relatively small, suggests the dominance of diffusion creep [50–52]. This is also confirmed by the relatively small grain sizes in the lower mantle (<∼1 mm) based on the grain growth laws for water-bearing conditions obtained here and for dry conditions determined previously [30]. Thus, the viscosity (*η*) follows a power-law relationship with grain size (*η*∝*d*^2^ for diffusion creep in Coble creep regime and *η*∝*d*^3^ for Nabarro–Herring creep regime) [53–55]. Consequently, the significantly larger grain sizes are expected to result in a much higher viscosity of LLVPs than the surrounding mantle, even though the temperature and water content within the LLVPs is elevated.

As explained above, estimating the absolute value of grain sizes in LLVPs and surrounding mantle may contain large uncertainty due to the unknown pressure dependence of grain growth rate. It is therefore unclear whether Coble or Nabarro–Herring creep dominates since they have different grain-size dependences. In particular, pressure may prohibit the grain growth rate significantly (for example the case of olivine [56]), leading to the preference of Coble creep in deeper regions despite the dominance of Nabarro–Herring creep at the shallow part of lower mantle [21,55]. We therefore compared the viscosity contrast of LLVPs and surrounding mantle in both Coble and Nabarro–Herring creep regimes.

Diffusion creep is controlled by diffusion of the slowest species along the fastest path [57–59]. In Bridgmanite, Si is among the slowest major diffusing species and therefore the creep rate is considered to be linearly proportional to the diffusion coefficient of Si (*D*_Si_) along the fastest path [40,41,60], i.e. Coble creep dominates when grain boundaries provide the most effective path, while Nabarro–Herring creep dominates when grain interior diffusion dominates. Thus, in the Coble diffusion creep regime [53], the viscosity is inversely proportional to $\delta D_{{\mathrm{Si}}}^{{\mathrm{gb}}}$ and proportional to the cube of the grain size (*d*),


(5) \begin{eqnarray*}
\eta \propto {d}^3T/\left( {\delta D_{{\mathrm{Si}}}^{{\mathrm{gb}}}} \right),
\end{eqnarray*}

where $D_{{\mathrm{Si}}}^{{\mathrm{gb}}}$ is the grain-boundary-diffusion rate of Si and δ is the grain-boundary width. The $\delta D_{{\mathrm{Si}}}^{{\mathrm{gb}}}$ term has been determined experimentally as a function of temperature [40].

Similarly, in the Nabarro–Herring diffusion creep regime [54], we have,


(6) \begin{eqnarray*}
\eta \propto {d}^2T/\left( {D_{{\mathrm{Si}}}^{{\mathrm{lat}}}} \right),
\end{eqnarray*}

where $D_{{\mathrm{Si}}}^{{\mathrm{lat}}}$ is the lattice diffusion of Si determined experimentally [40,41,60].

The atomic diffusivity of Si in a cation-vacancy mechanism is controlled by the concentration ([V_Si_]) and mobility (*D*_V_) of vacancies, i.e. *D*_Si_ = [V_Si_] × *D*_V_ [42,61]. With increasing temperature, both [V_Si_] and *D*_V_ increase due to thermal activation and therefore *D*^lat^ and δ*D*^gb^ increase [40]. Meanwhile *d* also increases since the grain growth rate increases with temperature [30]. On the other hand, ferric iron enhances the grain growth of bridgmanite [32], while its effect on the viscosity is relatively small [22,62]. Additionally, the incorporation of water largely enhances the grain growth of bridgmanite as demonstrated in this study. Because the water effects on the *D*^lat^ and *D*^gb^ of bridgmanite are unknown, we use the experimentally determined water effect of olivine [63,64] as a possible estimate of hydrous weakening.

**Figure 4.**
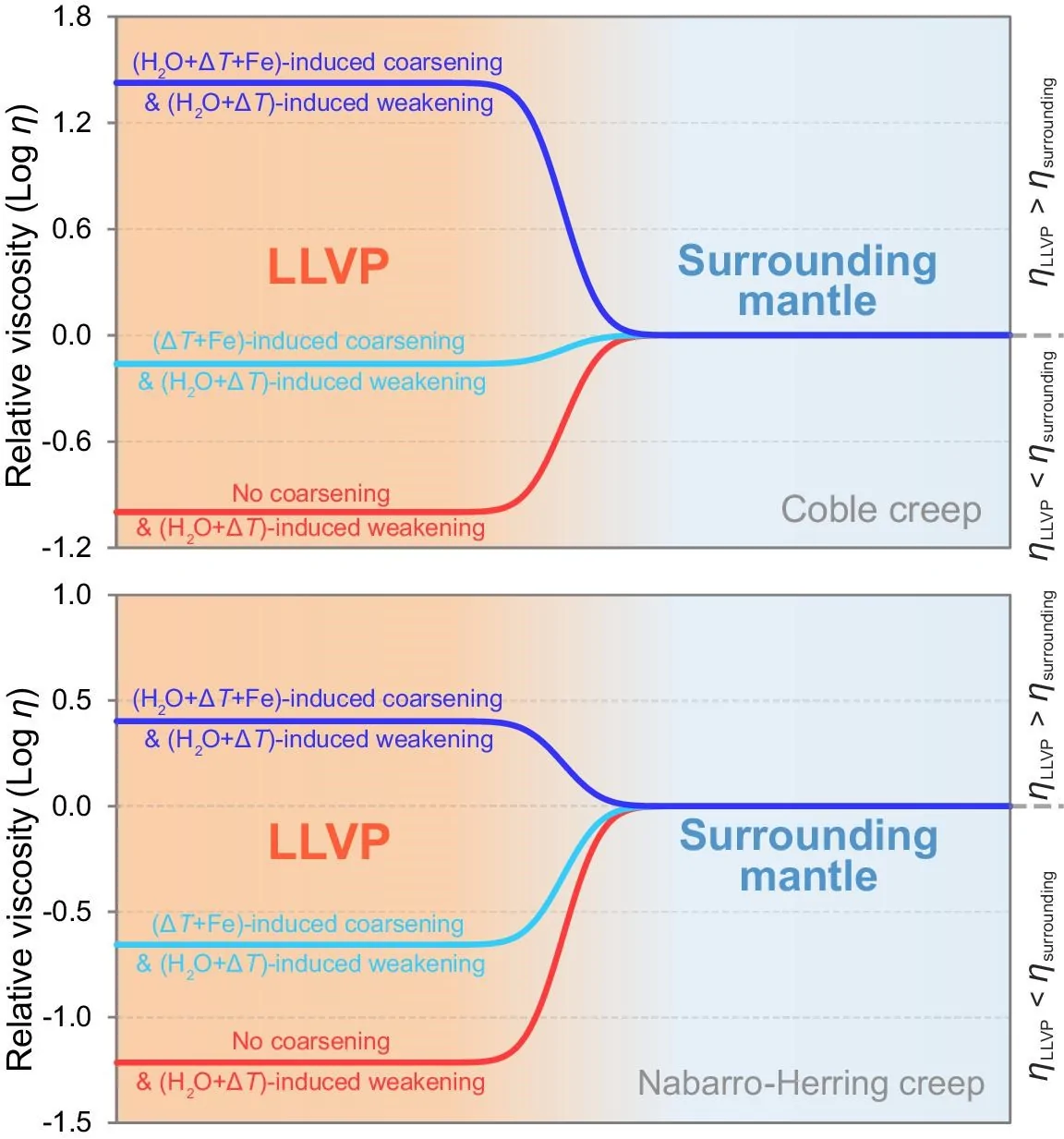
The viscosity contrast between the LLVPs and surrounding mantle. The vertical axis represents the relative viscosity of the LLVPs compared to the surrounding mantle (*η*_LLVPs_/*η*_surrounding_) in log scale calculated from the diffusion creep model in the Coble and Nabarro–Herring regimes [53–55] using the grain size of bridgmanite after a growth period of 100 Myr. The left and right parts of the figure represent the viscosity of the LLVPs and surrounding mantle, respectively. The curves labeled with (H_2_O + Δ*T*)-induced weakening indicate the viscosity contrast between LLVPs and surrounding mantle caused by the temperature difference (300 K) [14,15] and water contrast (1.6 orders of magnitude) [16,46,47] without considering the grain-size effects. When these weakening effects are included, those labeled with (Δ*T* + Fe)-induced coarsening and (H_2_O + Δ*T* + Fe)-induced coarsening represent the viscosity contrast by taking the temperature (Δ*T* = +300 K) [14,15], Fe^3+^ (10 mol.%) [10] and water (1.6 orders of magnitude) [16,46,47] enhancements of grain growth into account.

**Figure 5.**
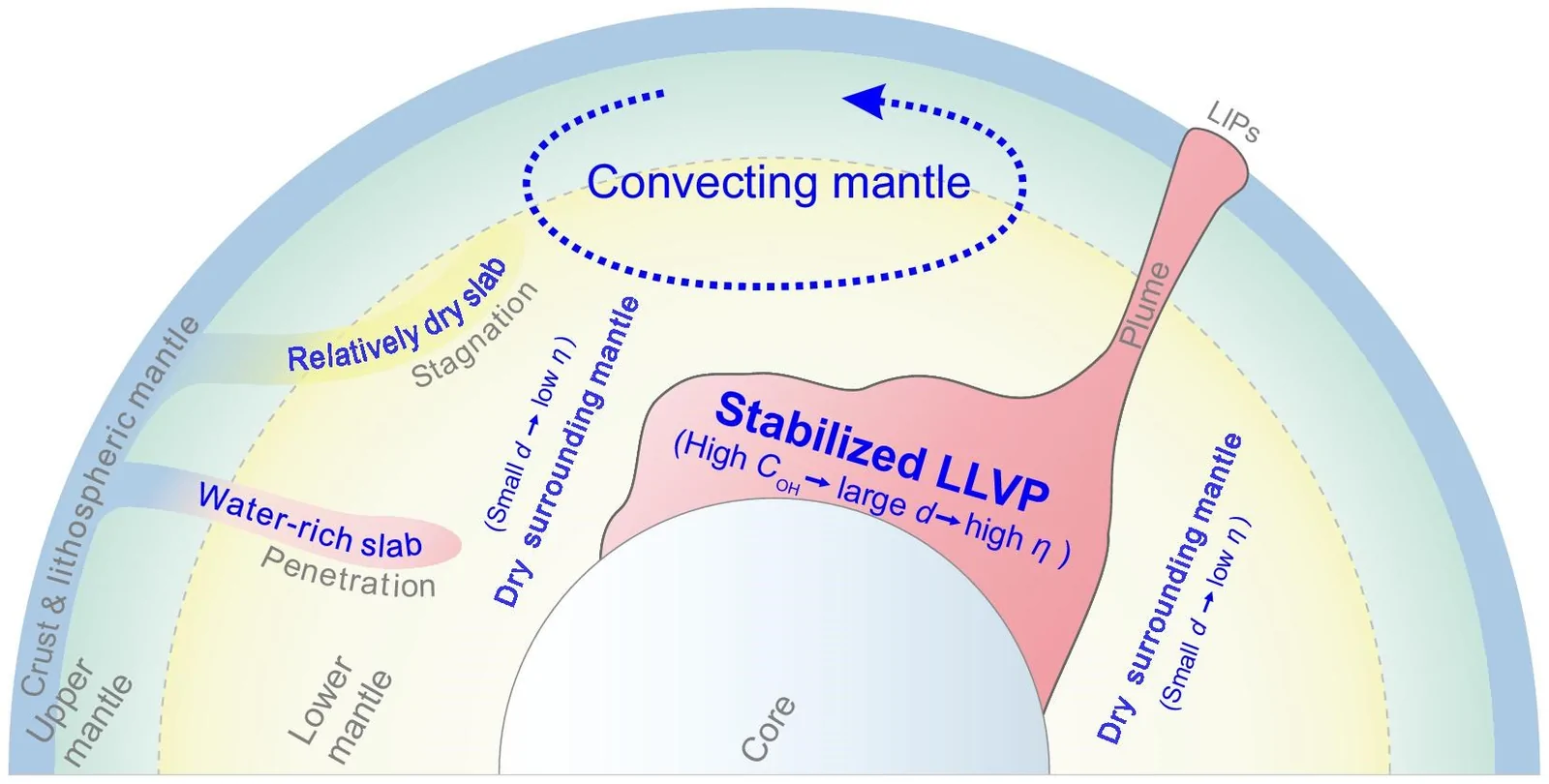
A schematic model of mantle geodynamics. The enhancement of grain growth by water increases the viscosity of LLVPs, leading to their stability over geological periods [3,6,7]. In contrast, the viscosity of the surrounding materials is limited due to the small grain sizes, resulting in the convection of the surrounding mantle. Meanwhile, the limited grain growth in some relatively dry slabs leads to their relatively low viscosity and thus stagnate at the topmost lower mantle, whereas water-rich slabs may develop large grain sizes with high viscosity and penetrate deeper without pronounced stagnation or folding [76].

Finally, we found that when only the weakening effects of elevated temperature and water are considered, the viscosity of LLVPs is predicted to be markedly lower than that of the surrounding mantle (e.g. Coble creep region in Fig. 3d). By further considering the grain-size effect, the viscosity ratio *η*_LLVPs_/*η*_surrounding_ increases significantly in both the Coble and Nabarro–Herring creep regimes, as calculated using Equations (5) and (6), respectively (Fig. 3e and f). As shown in Fig. 4, the elevated temperature and water content will cause the viscosity of LLVPs 1.0–1.2 orders of magnitude smaller than the surrounding mantle. However, by considering the grain growth enhancements by the high temperature [14,15] and the high iron content [10], the viscosity of LLVPs increases moderately but remain lower than that of the surrounding mantle, while by adding the water enhancement, the LLVPs have a viscosity up to 1.4 orders of magnitude higher than the surrounding mantle (Fig. 4). This viscosity contrast is expected to be sufficient to prevent LLVPs from being mixed by mantle convection [65,66], thus, enabling the long-term stability of LLVPs at the bottom of the lower mantle over billions of years [3,6,7] (Fig. 5). Although adding water may reduce the density of LLVPs, its effect should be negligible because the water content is at wt. ppm levels [34–36,43]. Additionally, bridgmanite to post-perovskite phase transition may occur at the bottom of the lower mantle, which may affect the viscosity contrast between LLVPs and surrounding mantle because post-perovskite is rheologically weaker than bridgmanite [67]. However, this phase transition is not expected to significantly affect grain size since post-perovskite will grow rapidly to the size close to that before the phase transformation [68] Meanwhile, the positive Clapeyron slope [69] for the phase transition suggests more post-perovskite in the surrounding mantle than LLVPs, which would further enhance the viscosity contrast [67].

Moreover, the *D*^lat^ and *D*^gb^ may have different pressure dependences and therefore the dominance of creep mechanism may change with pressure between Nabarro–Herring creep and Coble creep because they are controlled by *D*^lat^ and *D*^gb^, respectively. However, the viscosity ratio *η*_LLVPs_/*η*_surrounding_ in either Coble or Nabarro–Herring creep regime should be independent from pressure by assuming the same pressure dependence of grain growth rate and same creep mechanism in LLVPs and surrounding mantle. In case if the LLVPs are governed by Nabarro–Herring creep while the surrounding mantle is deformed by Coble creep, the *η*_LLVPs_/*η*_surrounding_ will be lower than that for full Coble creep dominated, but higher than that for full Nabarro–Herring creep dominated. Namely, the both-Coble-creep model (Fig. 4a) and both-Nabarro–Herring creep model (Fig. 4b) for LLVPs and surrounding mantle give the upper bound and lower bound, respectively, of the viscosity contrast.

Note that if water has a weaker effect on Si diffusivity in bridgmanite than in olivine, the water weakening on bridgmanite strength would be smaller and thus the viscosity contrast between LLVPs and surrounding mantle would be further enhanced. Conversely, if water has a stronger effect on bridgmanite diffusivity, the net viscosity contrast would be reduced. Although the viscosity contrast calculation is subject to this uncertainty, the central conclusion remains that water-induced bridgmanite coarsening provides a viable mechanism for increasing LLVP viscosity.

The grain-size-induced high viscosity of LLVPs reconciles the spatial correlation of the reconstructed locations of hotspots and LIPs with the present-day configurations of LLVPs [6,7]. It also effectively preserves ancient materials within LLVPs, accounting for the distinct primitive isotopic signatures observed in hotspot lavas [70,71]. In addition, mantle flow along the margins of LLVPs with a pronounced rheological contrast can explain the observed localized seismic anisotropy [72]. The related sharp edges of LLVPs (strong lateral δV_S_ gradients) [73] may account for the initialization of mantle plumes on the LLSVP margins [74]. Furthermore, the grain-size contrast also aligns with attenuation tomography results, which show that LLVPs are less attenuating than the surrounding mantle [2]. While their low seismic velocities refer to elevated temperatures, low attenuation is attributed to coarser grain sizes since water alone does not decrease attenuation [75].

The significant water-induced coarsening of bridgmanite may also provide new insights for slab dynamics in the lower mantle (Fig. 5). Variations in water content among different subducted slabs lead to their distinct grain sizes and therefore rheological behaviors. The grain growth in relatively dry slabs should be limited, leading to their relatively low viscosity and thus stagnate at the topmost lower mantle [30]. In contrast, more water-rich slabs may have developed large grain sizes, allowing them to penetrate into deep regions easily without stagnation or folding. Such rheological contrasts provide a plausible explanation for the variable slab subduction depths and geometries [76].

## METHODS

### Starting materials and capsule preparation

San Carlos olivine powder with particle sizes of 1–10 μm grounded from single crystals were used as starting materials. A powder mixture of MgO and Mg(OH)_2_ (50:1 weight ratio) was prepared as a water source. After drying in a vacuum furnace at 400 K, three layers of powders, i.e. water source, olivine powder and MgO powder, separated by Pt foils, were loaded into Pt capsules (Fig. S1) and sealed by welding. The diameter of the Pt foils (0.7 mm) was slightly smaller than the inner diameter of the capsule (0.8 mm) so that water could migrate freely within the capsule. The MgO layer was used to examine the homogeneity of the water distribution within the capsule. The amount of water source was minimized to prevent the formation of hydrous melt. The bulk water contents in the capsules were roughly controlled by the amount of water sources (thickness of the water source layer), which may have large uncertainty because even small amounts of water source (such as 0.1–0.2 mm thickness) will cause saturation.

### Grain growth experiments

High-pressure experiments were performed using a 1000-ton Kawai-type multi-anvil press with 7/3 cell assemblies. Each assembly was compressed to 25 GPa, followed by heating at 1500 K for 1 h monitored by a D-type (W/Re) thermocouple. This is sufficient for the phase transformation of olivine to bridgmanite + ferropericlase [77], the decomposition of Mg(OH)_2_ from the water source layer to MgO + H_2_O and the uniform distribution of H_2_O in the samples [78,79]. Subsequently, the temperature was raised up to 2000 K and maintained for 10–500 min for grain growth. Finally, the assembly was quenched to room temperature by cutting off the heating power and decompressed to ambient conditions for >15 h.

Note that the grain size of the samples is reduced to theoretical zero by the phase transition from olivine to bridgmanite + ferropericlase, followed by grain growth at 1500 K and finally growth at 2000 K. Although the presence of water may affect the growth rate at 1500 K and thus affect *d*_0_ in Equation (1), the grain growth at 1500 K should be negligible compared to that at 2000 K based on the activation enthalpy reported previously [30]. Therefore, we have *d*_t_ >> *d*_0_ in Equation (1).

Since the bulk water contents in the sample capsules are extremely low (less than a few thousand wt. ppm), it is technically difficult to quantify the potential variation of water content (and water fugacity) with experimental duration. However, both the decomposition of Mg(OH)_2_ and the water diffusion along grain boundaries are extremely fast [78,79]. As far as water is equilibrated in the sample, the water content should remain constant as confirmed by similar experiments for olivine [63,64].

### Statistical analysis of bridgmanite grain size

BSE images were taken on the polished cross sections of the recovered samples using a JSM-IT800 scanning electron microscope equipped with a field emission gun operated at an accelerating voltage of 15 kV. The grain boundaries of bridgmanite were manually traced in the BSE images, and the area of each grain was obtained using image processing software (ImageJ, https://imagej.net). The grain size was determined by the equivalent circular diameter method with a 3D correction factor of 1.56 (see ref. [80]). For each sample, grain-size analysis was performed on BSE images taken in different regions, totaling >300 bridgmanite grains (Fig. S2).

### Electron probe analysis

The chemical compositions of bridgmanite were determined using a JEOL JXA-iHP200F field-emission EPMA. Analyses were performed using a focused beam with an accelerating voltage of 15 kV and a current of 15 nA, with a counting time of 20 s for each spot. Forsterite and metallic iron were used as standards for Mg, Si and Fe, respectively. The EPMA results of bridgmanite are given in Table S2, showing nearly identical compositions (iron contents) in different samples. Therefore, although the compositions of ferropericlase were not analyzed due to their small grain sizes, they are expected to be identical as well by assuming a constant iron partition coefficient. Thus, the log*d* versus water content relationship in Fig. 2 should not be affected by compositional variation.

### Infrared spectrometer analysis

The sample capsules were polished on both sides to a thickness of ∼100 μm and >4 unpolarized infrared spectra were taken on the periclase phase (from both the MgO and MgO + Mg(OH)_2_ layers) of each capsule using a Bruker VERTEX70v spectrometer equipped with a Hyperion II infrared microscope. For each spectrum, 128 scans were accumulated with a resolution of 4 cm^−1^. The aperture size was 40 × 40 to 100 × 100 μm^2^ depending on the sample sizes.

The infrared spectra showed typical absorption peaks of periclase at ∼3297, ∼3309 and ∼3372 cm^−1^ (see refs [81,82]) (Figs S4 and S5). Water content in minerals is generally calculated as the product of a characteristic absorption coefficient and the integrated area of the infrared peaks (total absorptions) according to the Beer–Lambert law. However, because the absorption coefficient of periclase is unknown and our focus is on relative change of water content rather than the absolute values, we use the total integrated peak area as a proxy for its water content.

Therefore, after a leveling of the initial spectra for visibility of the absorption bands (Fig. S5a, and b), a baseline correction (Fig. S5c) and thickness normalization to 1 cm (Fig. S4), the total infrared absorptions (${A}_{{\mathrm{OH}}}$) in periclase were calculated as the integral area of the absorption peaks,


(7) \begin{eqnarray*}
{A}_{{\mathrm{OH}}} = \int K\left( { \!\!\!\bar {\,\,\, v} } \right)d \!\!\!\bar {\,\,\, v} ,
\end{eqnarray*}

where $K(\!\!\!\bar {\,\,\, v})$ is the absorption at a wavenumber $\!\!\!\bar {\,\,\, v}$ (Fig. S4).

The approximate estimates of water contents in periclase based on the Paterson (1982) calibration were given in Table S1 as a reference, while those in bridgmanite were not calibrated due to the lack of reliable absorption peaks.

## Supplementary Material

nwag464_Supplemental_File

## Data Availability

The data supporting this study are available in Zenodo at https://doi.org/10.5281/zenodo.21799660.
