# Supplementary material for: Water-induced bridgmanite coarsening may produce highly viscous lower mantle seismic anomalies
Source: Natl Sci Rev. 2026 Jul 31;13(16):nwag464. doi: 10.1093/nsr/nwag464 (PMC13547607; doi:10.1093/nsr/nwag464)
Supplement: Supplementary file 1 [file nwag464_supplemental_file.pdf]

Supplementary Materials for

Water-induced bridgmanite coarsening may produce highly-viscous lower mantle seismic anomalies

Yifu Lyu[1], Hongzhan Fei[1*], Amrita Chakraborti[2,3], Maxim Ballmer[4], Ulrich Faul[5], Shan Zhou[1], Yan Yang[1], Jia Liu[1], Fang Xu[1], Suyu Fu[1], Baohua Zhang[1], Qunke Xia[1*], Tomoo Katsura[2]

1. Research Center for Earth and Planetary Material Sciences, School of Earth Sciences, Zhejiang University, Hangzhou 310058, China
2. Bayerisches Geoinstitut, University of Bayreuth, Bayreuth 95440, Germany
3. Unité Matériaux et Transformations, Université de Lille, Lille 59000, France
4. Department of Earth Sciences, University College London, London WC1E 6BT, UK
5. Earth Atmospheric and Planetary Sciences, Massachusetts Institute of Technology, Cambridge, MA 02139, USA

Corresponding authors: H. Fei (feihongzhan@zju.edu.cn), Q. Xia (qkxia@zju.edu.cn)

This PDF file includes:

Figure. S1 to S6

Tables S1 to S2

References

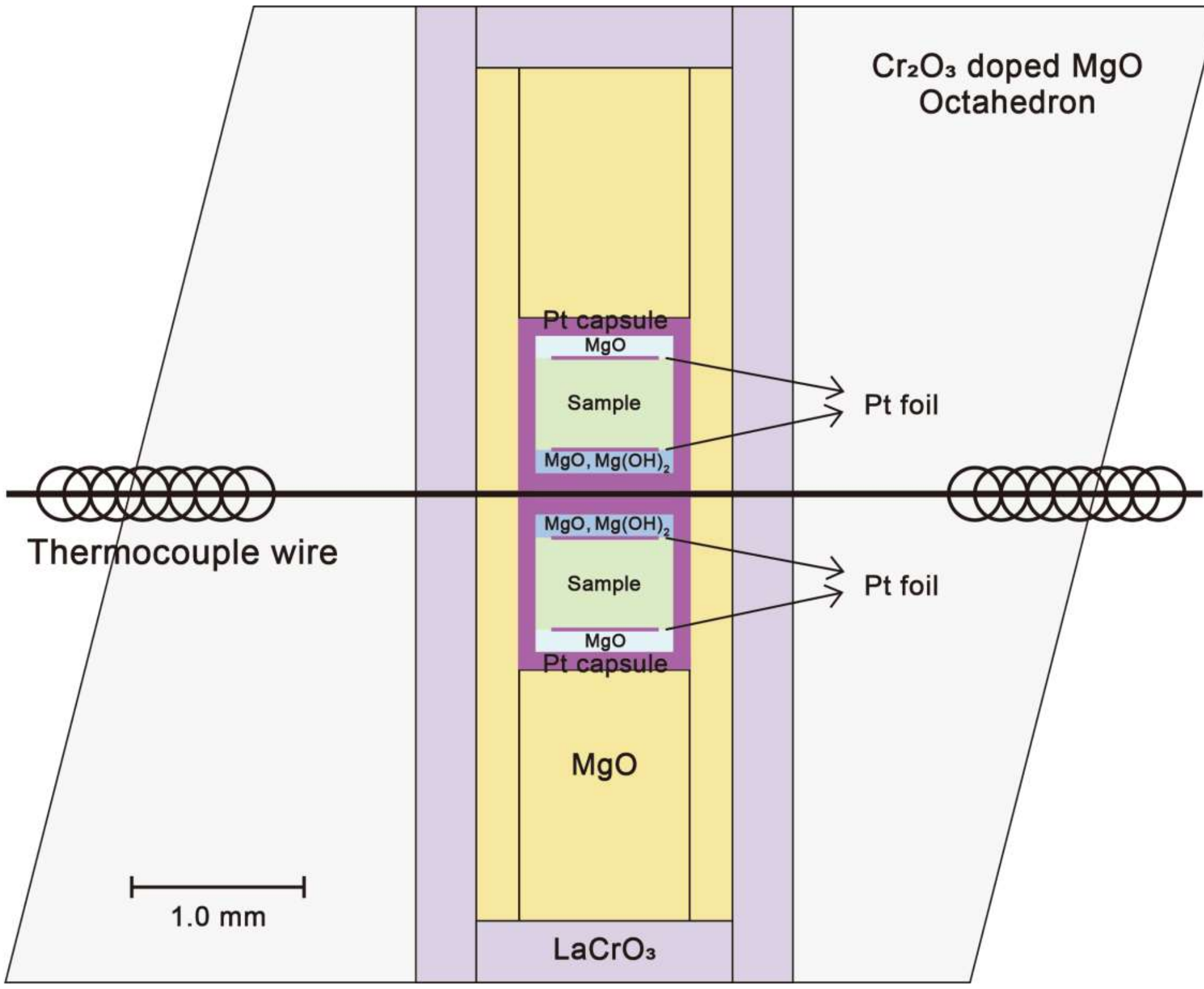

Figure. S1. Cell assembly design for the grain growth experiments. Each Pt capsule contains three layers of powder (MgO+$Mg(OH)_2$ water source, olivine sample, and MgO powder) separated by Pt foils. The diameter of the Pt foils is slightly smaller than the inner diameter of the capsule to guarantee the homogenization of water freely within the capsule.

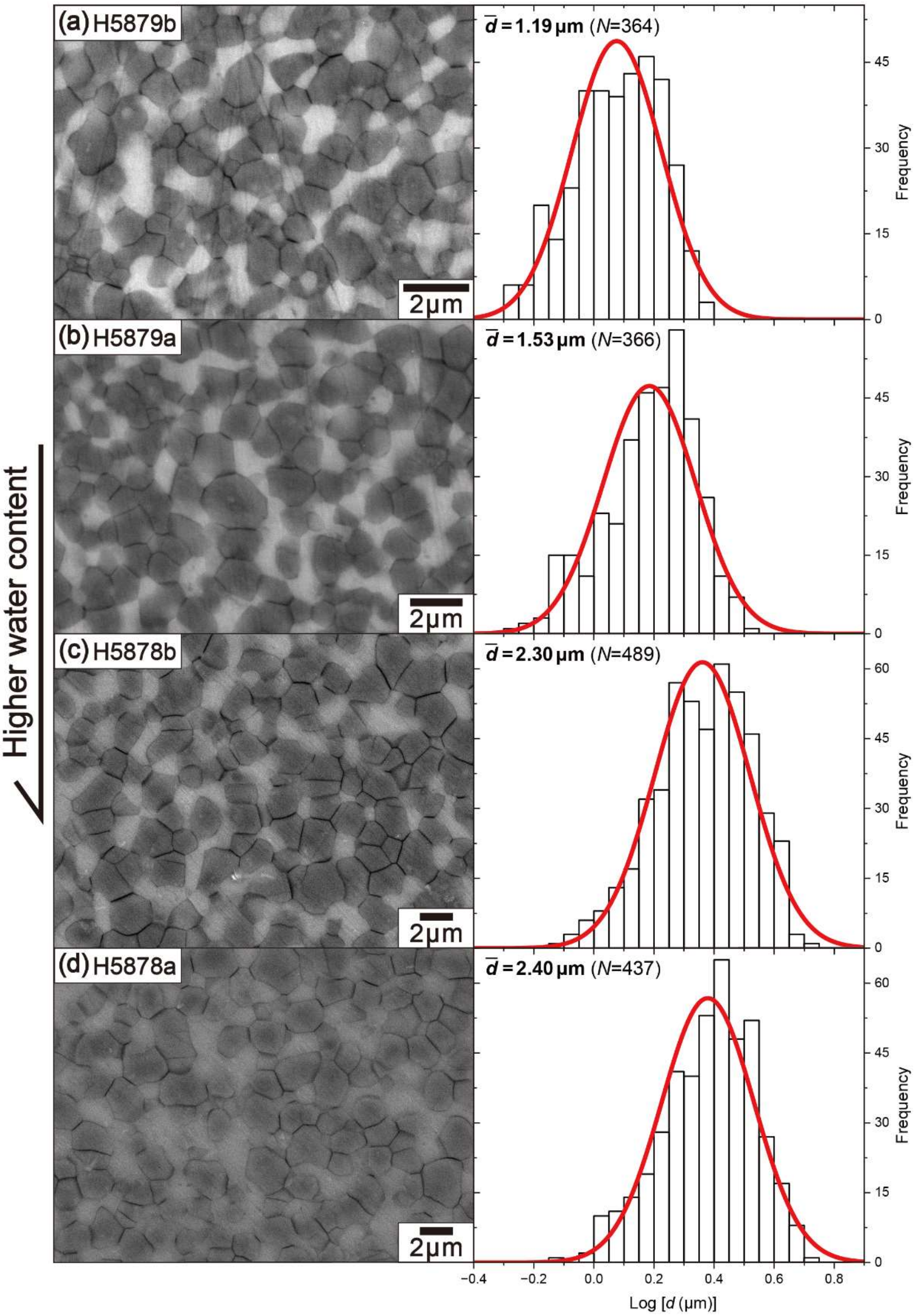

Figure. S2. Grain size of bridgmanite increases systematically with increasing relative water content. Left: BSE images. Right: grain size distributions. All samples were obtained under the same pressure (25 GPa) and temperature (2000 K) conditions and the same growth durations (500 minutes) but different water contents (Table S1). $\overline{d}$: the average grain size of bridgmanite. *N*: number of grains.

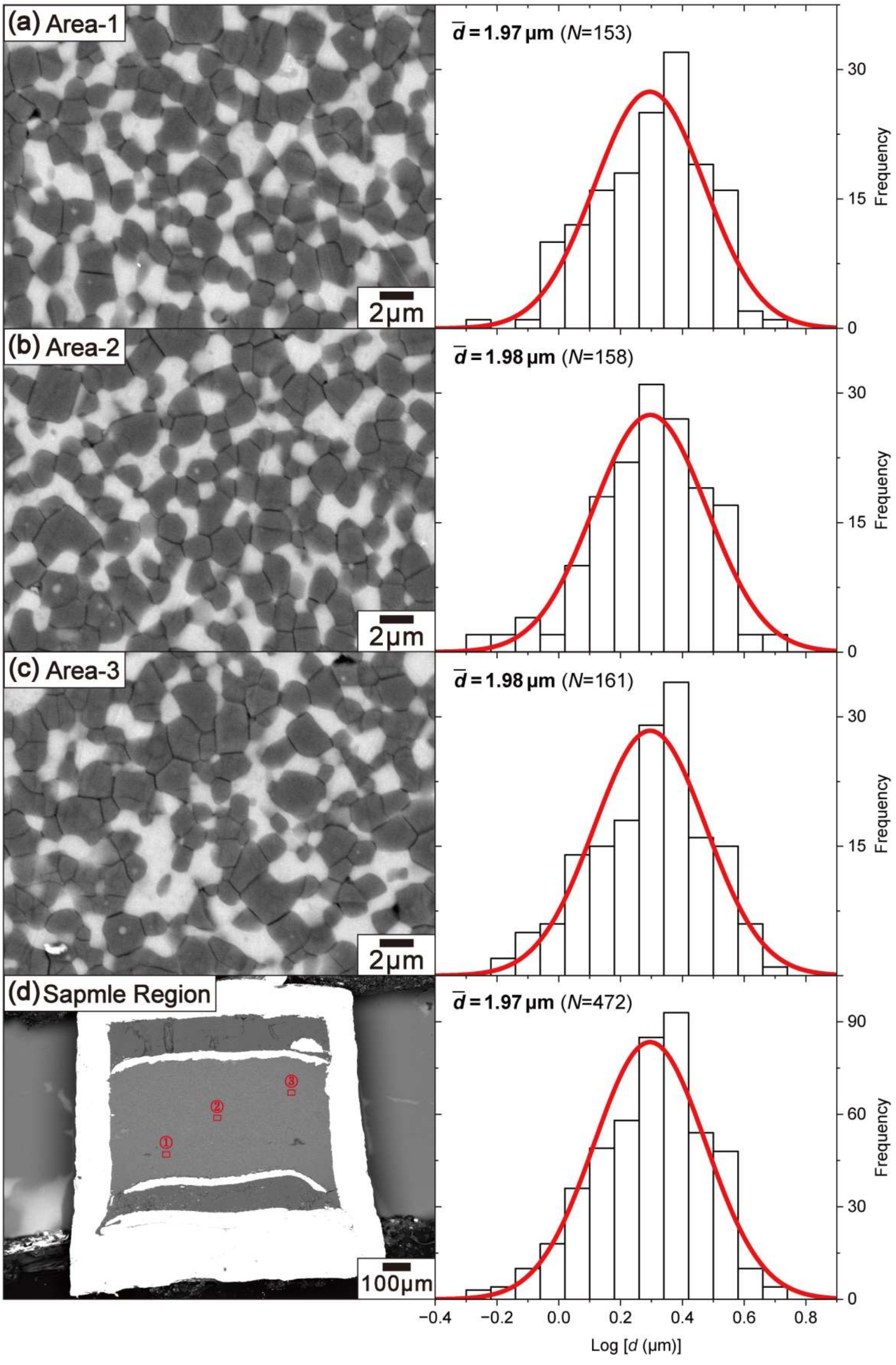

Figure. S3. Uniform grain size distribution of bridgmanite from different regions of the sample (H5884a, 25 GPa, 2000 K, 30 min). Left: BSE images. Right: grain size distribution. $\bar{d}$: the average grain size of bridgmanite. *N*: number of grains. The red boxes in panel d correspond to the regions in panels a, b, and c, respectively, and the statistical result in panel d summarizes those from panels a–c.

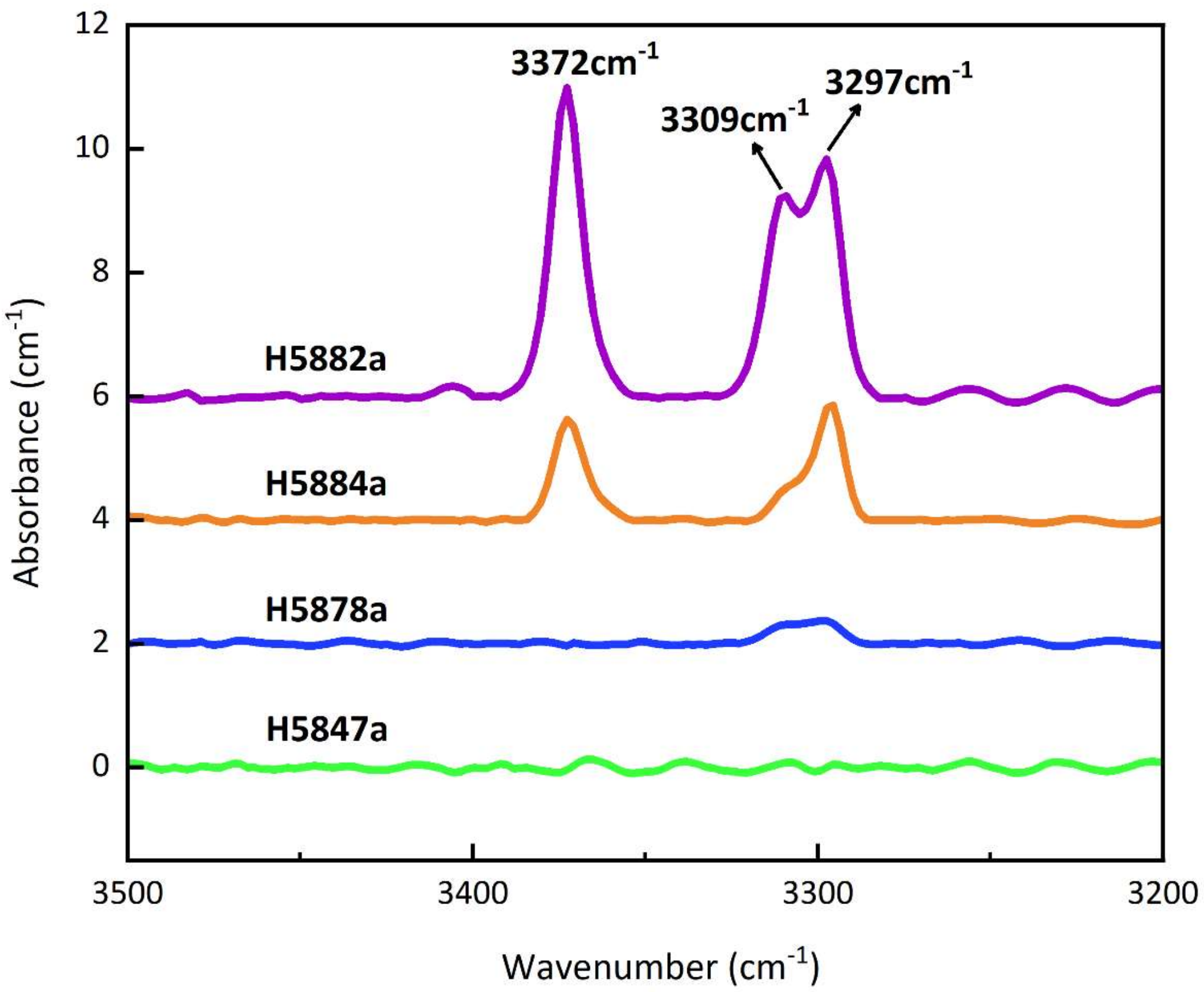

Figure. S4. Infrared spectra of MgO with various absorptions. The marked absorption bands in the spectra correspond to distinct OH infrared bands in MgO at wavenumbers of 3297, 3309, and 3372 cm^{-1}.

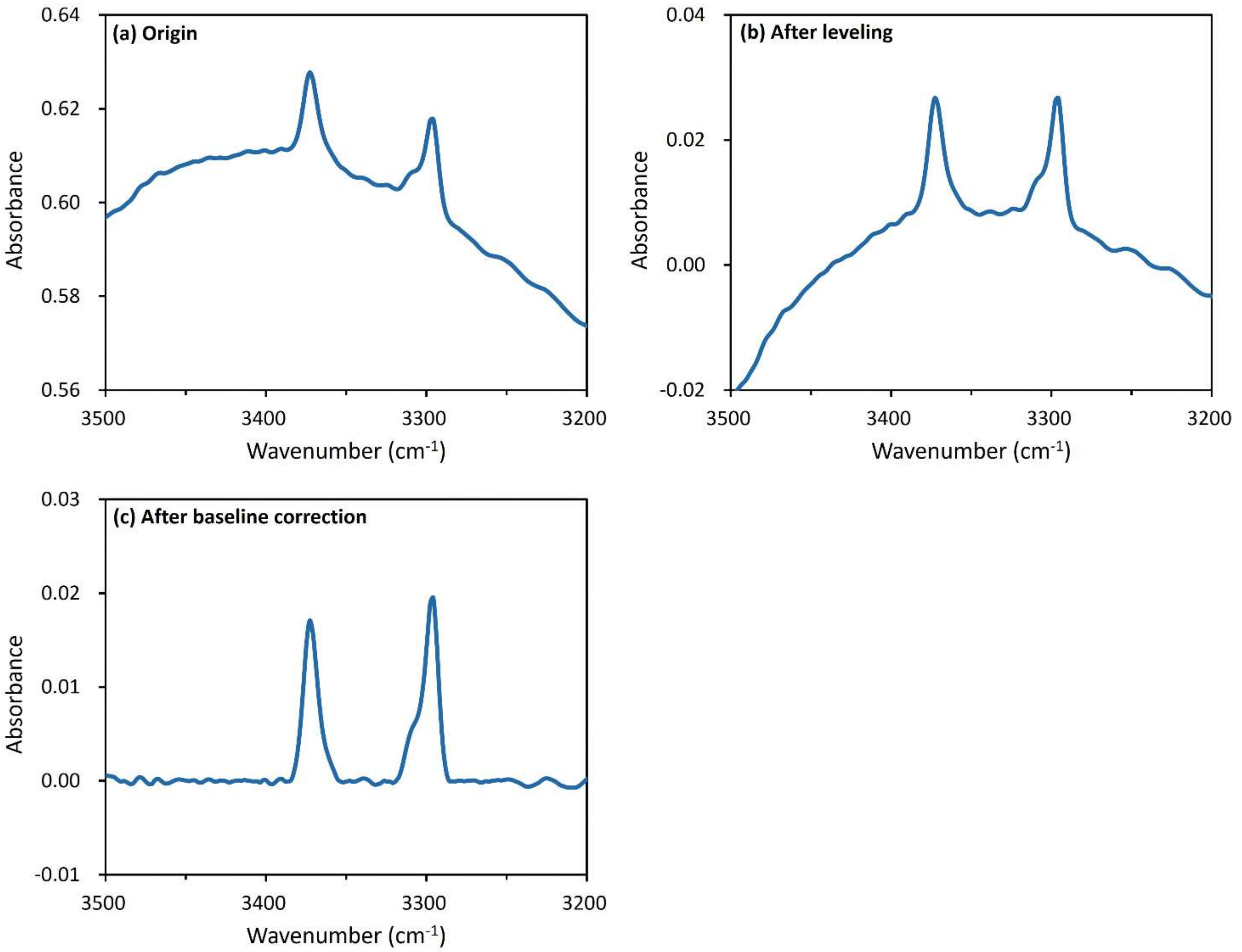

Figure. S5. Representative FTIR spectra (Run H5884a) showing infrared bands in MgO. (a) Raw FTIR spectra; (b) leveled FTIR spectra; (c) baseline-corrected FTIR spectra.

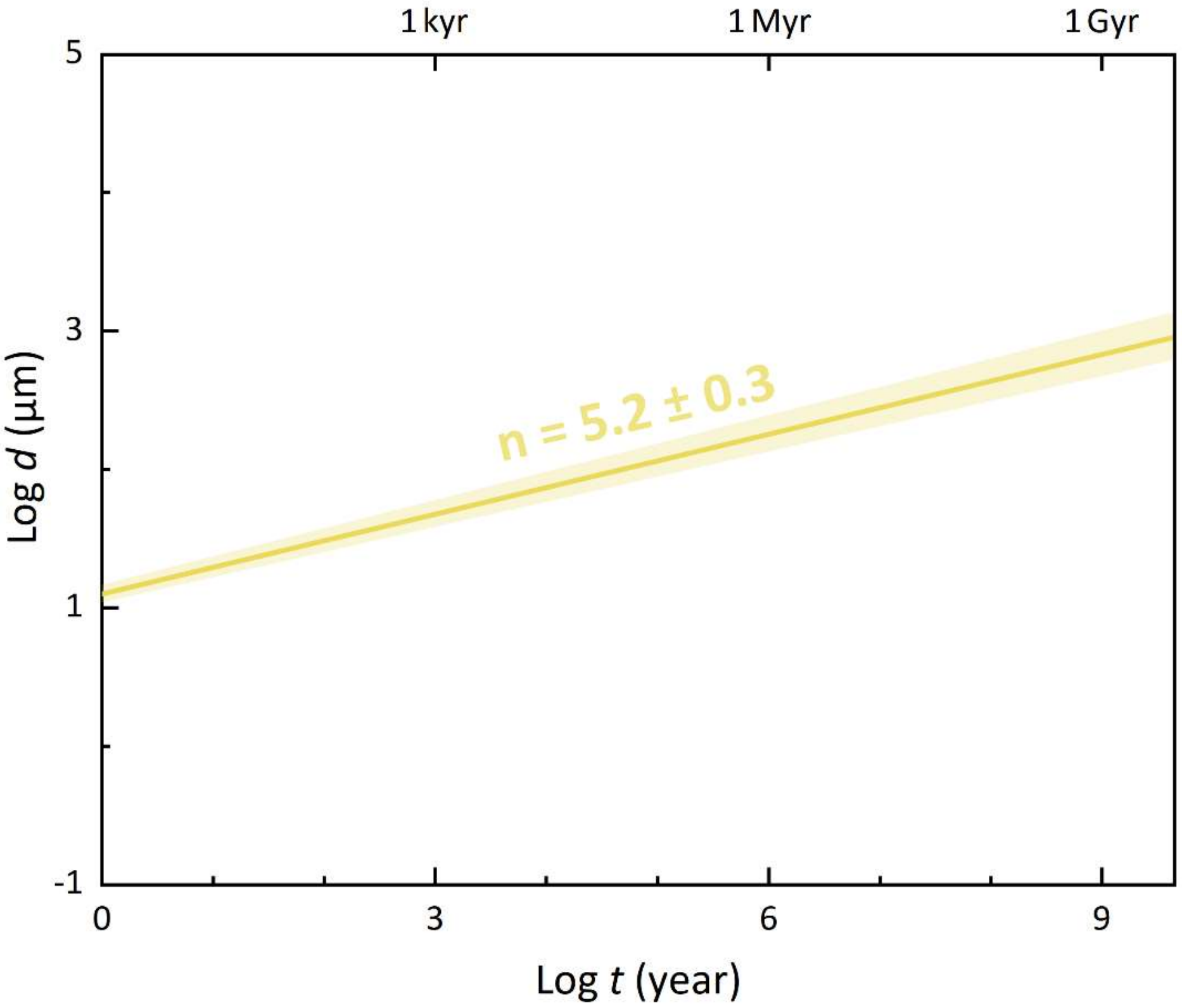

Figure. S6. Long-time extrapolation of grain size. Grain size is extrapolated using the grain growth exponent n = 5.2 ± 0.3 [1], assuming growth starts at log *t* (min) = 0 with log *d* (μm) = 0. The shaded band denotes the 1σ uncertainty of the extrapolation.

Table. S1. Experimental conditions, bridgmanite grain size (*d*), grain growth rate constant (*k*), total infrared absorptions in MgO (A_{OH}), and approximate water content in MgO (C_{OH}).

Run No.*a	*t* (min)	Analysed grains	Log *d* (μm)	*d* (μm)	*k* ($\mu m^n/s$)*b	Log A_{OH} (cm^{-2})*c	A_{OH} (cm^{-2})	Log C_{OH} (wt. ppm)*e
H5847a	100	579	-0.25 (13)	0.56	8.14×10^{-6}	--*d	--*d	--*d
H5847b		479	0.14 (16)	1.38	8.81×10^{-4}	0.56 (9)	3.63	-0.42 (9)
H5878a	500	437	0.38 (15)	2.39	3.12×10^{-3}	0.78 (7)	6.03	-0.20 (7)
H5878b		489	0.36 (16)	2.30	2.52×10^{-3}	0.75 (17)	5.62	-0.28 (16)
H5879a	500	366	0.18 (15)	1.53	3.04×10^{-4}	0.41 (11)	2.57	-0.56 (11)
H5879b		364	0.08 (15)	1.19	8.27×10^{-5}	0.24 (17)	1.74	-0.74 (17)
H5882a	10	384	0.49 (23)	3.07	5.70×10^{-1}	2.17 (5)	147.91	1.22 (5)
H5884a	30	472	0.30 (18)	1.97	1.91×10^{-2}	1.60 (15)	39.81	0.64 (15)
H5884b		514	0.15 (17)	1.41	3.32×10^{-3}	1.37 (11)	23.44	0.42 (11)
H5885b	500	447	0.12 (17)	1.31	1.38×10^{-4}	0.33 (3)	2.14	-0.63 (3)

*a: Each run contains two capsules, labeled a and b, respectively.

*b: The grain growth rate *k* was calculated with the grain growth size constant $n = 5.2 \pm 0.3$ (ref. [1]).

*c: The A_{OH} refers to the total infrared absorptions in MgO. It may have relatively large uncertainty when the absorptions are small due to the limited peak heights.

*d: The starting material in this capsule did not contain $Mg(OH)_2$, while no absorption bands were detected by infrared analysis.

*e: The C_{OH} refers to an approximate estimate of the absolute water content in MgO and is provided for reference only. We apply the Paterson (1982) calibration [2]: $C_{\mathrm{OH}}^{\mathrm{MgO}} = \frac{X_i}{150\zeta}\int\frac{K(\bar{v})}{(3780-\bar{v})}d\bar{v}$, where X_i is the density factor ($X_i = 2573$ for periclase), ζ is the orientation factor ($\zeta = 1/3$), and $K(\bar{v})$ is the absorbance at wavenumber $\bar{v}$.

Table. S2. Composition of bridgmanite analyzed by EPMA. The weight percentages are calculated by assuming all iron as ferrous. *N*: number of point analyses.

Sample	*N*	MgO (wt.%)	SiO_2 (wt.%)	FeO (wt.%)	Total (wt.%)	Mg (atomic)	Si (atomic)	Fe (atomic)	Fe/(Mg+Fe) ratio (%)
H5878a	17	38.65 (115)	59.47 (124)	2.44 (58)	100.56 (155)	7.74 (16)	7.99 (8)	0.28 (7)	3.43 (85)
H5878b	15	38.19 (116)	59.96 (107)	2.64 (29)	100.79 (161)	7.63 (16)	8.04 (7)	0.30 (4)	3.74 (48)
H5882a	19	39.20 (87)	59.12 (102)	2.45 (50)	100.77 (125)	7.58 (14)	7.94 (7)	0.28 (6)	3.39 (70)
H5884a	13	38.57 (93)	58.94 (96)	2.53 (31)	100.04 (128)	7.77 (16)	7.97 (9)	0.29 (3)	3.55 (39)

Note that not all samples were analyzed by EPMA because of their relatively small grain sizes, thus, the compositional analyses were significantly affected by signals from adjacent phases.

References

1. Fei H, Faul U, Katsura T. The grain growth kinetics of bridgmanite at the topmost lower mantle. *Earth Planet Sci Lett* 2021; **561**: 116820.

2. Paterson MS. The determination of hydroxyl by infrared absorption in quartz, silicate glasses and similar materials. *Bull Minéral* 1982; **105**: 20–9.
